# Asymmetrical effects of real exchange rate on the money demand in Saudi Arabia: A non-linear ARDL approach

**DOI:** 10.1371/journal.pone.0207598

**Published:** 2018-11-28

**Authors:** Haider Mahmood, Tarek Tawfik Yousef Alkhateeb

**Affiliations:** 1 Department of Finance, College of Business Administration, Prince Sattam bin Abdulaziz University, Al-Kharj, Saudi Arabia; 2 Department of Marketing, College of Business Administration, Prince Sattam bin Abdulaziz University, Al-Kharj, Saudi Arabia; 3 Kafrelsheikh University, Kafrelsheikh, Egypt; Central South University, CHINA

## Abstract

This present research investigates the money demand function of Saudi Arabia using a long period 1968–2016. In addition, the asymmetrical effects of real exchange rate changes have also been explored in the estimated money demand function. Our empirical results suggest that income and inflation have positive and negative effects on money demand respectively. Further, a real appreciation of US dollar has a positive effect but a real depreciation has a negative effect on the money demand. Furthermore, income and price homogeneity hypotheses do not hold for the estimated elasticities. Moreover, the estimated model is found stable with the theoretically expected effects of money demand’s determinants. Therefore, we are suggesting money supply as a monetary policy instrument to the economy of Saudi Arabia.

## Introduction

An estimation of Money Demand Function (MDF) carries a prime importance in the macroeconomic literature. It explains that how other sectors of an economy, such as real sector and financial sector, shape the demand for money and thus the monetary policy. The choice of monetary policy instrument also depends on the stability of MDF. Poole [1] suggested the money supply as monetary policy instrument in the presence of a stable MDF and the interest rate otherwise. The stability issue requires a precise choice of determinants of money demand. In which, exchange rate variable plays a great role in stabilizing the MDF [2,3]. At first, Mundell [4] proposed the exchange rate as a major determinant of money demand without an empirical support. Afterwards, a mass of literature has tested and reported the significant and insignificant effects of exchange rate on the money demand. Using data sets of different countries, some studies have corroborated that asymmetrical effects of exchange rate have been proved significant which was found insignificant by past literature in the symmetrical settings. These studies have also reported a stable MDF after including asymmetrical effects of exchange rate [5,6,7,8]. Therefore, symmetrical effect of exchange rate in the MDF may be alleged for a misspecification of model in the presence of significant asymmetry. The studies on the asymmetrical effects of exchange rate are very scant in the global monetary literature. Exchange rate has two types of movements, an appreciation and a depreciation, and it is not necessary that both movements have same magnitude or same direction of effects on the money demand.

The exchange rate may have positive or negative effect on the Money Demand. For example, an appreciation of a foreign currency increases the domestic value of foreign assets and people may sell the foreign assets/currency for a capital gain. Resultantly, local money demand may be increased. This relationship may be claimed for a wealth effect [9]. On the other hand, an appreciation of a foreign currency may develop the expectations of further appreciation. In this case, people may hold or buy more of the foreign currency in their portfolio for speculative purposes. Consequently, demand for local currency may be decreased and this relationship may be claimed for a substitution effect or an expectation hypothesis [7]. Therefore, a single event of a foreign currency appreciation may increase or decrease the local money demand and exact relationship is an empirical question.

**Fig 1:**
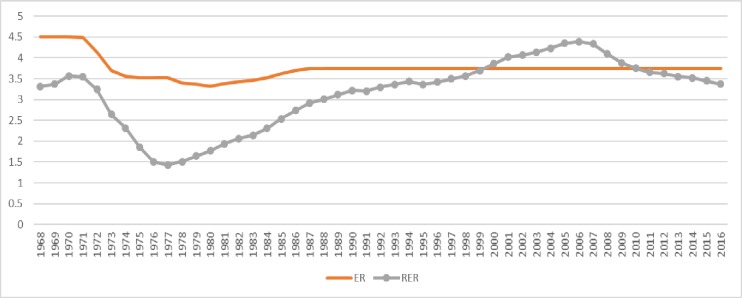
Trends of exchange rate (ER) and real exchange rate (RER) over a period 1968–2016.

Fig 1 shows the trends of ER and RER over a selected sample time period. ER is a bilateral nominal exchange rate and is defined as 1 US dollar = number of Saudi Arabian Riyals (SAR). RER is bilateral real exchange rate and ER is converted into RER by adjusting the inflation of USA and Saudi Arabia. Here, positive trends of ER and RER are showing appreciation of US dollar and vice versa. ER has been fixed at 1 US dollar = 4.5 SAR during 1968–1970 and at 3.75 during 1987–2016. A sharp decline in ER was observed during 1972–1974. Further, a sharp decline in RER has also been observed during 1972–1976. Otherwise, ER has slight fluctuations during 1975–1986. Further, RER has a long consecutive negative trend during the periods 1971–77 and 2007–2016. Conversely, it has a consistent positive trend during a period 1968–1970 and during a long period 1978–2006 except the minute declines in the years 1991 and 1995.

In the estimation of money demand function, we may find a vast literature available for a single country [10]. Therefore, we may focus on Saudi monetary literature to highlight the contribution of this research. At first, Darrat [11] investigated the money demand’s determinates for Saudi Arabia but he ignored the cointegration analysis which seems very important in the analysis of the MDF. Afterwards, a number of studies have applied cointergation tests on the money demand function of Saudi Arabia but ignore the exchange rate as a determinant [12,13,14,15] and some studies hypothesize the symmetrical effect of exchange rate on the money demand [16,17,9,18,19,20]. Both problems of ignoring exchange rate or assuming symmetrical effect of exchange rate may become the reasons for the misspecification and unstable MDF [10,5,6,8]. Consequently, some of the studies have reported the theoretically unexpected results for Saudi MDF. For example, positive effect of interest [19,15], insignificant effect of interest rate [11], insignificant effect of income [19] and positive effect of inflation [13] have been reported. Moreover, most of the studies assuming a symmetrical effect of exchange rate have reported the positive effect of exchange rate on money demand using the time periods 1977–1997 [17], 1979–2000 [18,19] and 1980–2014 [20,15]. These studies utilize the time periods in which a consistent real appreciation of US dollar was dominant. For example, a consistent real appreciation of US dollar has been observed during a period 1978–2006 except some minute depreciations in the years 1991 and 1995. Therefore, these studies have neglected either a consecutive real depreciation during a period 1971–1977 or 2007–2016 or both. On the other hand, Al Rasasi [16] corroborates a negative effect of exchange rate on money demand using a period 1993–2015 in which real exchange rate has a balance number of observations of both real depreciation and real appreciation. In the asymmetry analysis, Alsamara et al. [21] investigate the effects of oil price changes on the money demand by utilizing quarterly series of a period 1990–2014. They find the asymmetric effects of rising and falling oil prices on the money demand and report that positive shocks in oil price exhibit greater impact than that of negative shocks. Wen et al. [22] reports a nonlinear causality from oil price to US dollar exchange rate. So, asymmetric effects of exchange rate on Saudi money demand may also be expected.

Saudi Arabia with fixed-pegged exchange rate system requires a precise estimate of MDF including exchange rate in the analysis. Because, this country uses a lot of foreign currency reserves to keep the exchange rate stable and her monetary policy is also facing constraint due to keeping exchange rate fixed. Foreign currency reserves are utilized for a countercyclical reaction of any cyclical fluctuation in the global oil market due to a heavy dependence of Saudi economy on the oil export and oil price [23]. Moreover, oil price may also have a deep impact on the income, aggregate demand and liquidity of money. Consequently, the change in aggregate demand may also affect the inflation and real exchange rate. Any expected movement in exchange rate may accelerate the speculative and hedging activities [23] which may greatly affect the money demand in response. Although, Alsamara et al. [21] has tested the asymmetric direct effects of oil price, assuming it scale variable instead of income level, in the Saudi MDF. But, still asymmetric effects of real exchange rate are missing in the Saudi monetary literature as speculation activities may response differently to an event of appreciation or depreciation. Further, a mix evidence of a positive effect of exchange rate on Saudi money demand with dominant appreciation of US dollar in the limited sample time periods [17,18,19,20] and a negative effect of exchange rate with sufficiently large time sample [16] motivates us to test the asymmetric effects of real exchange rate on the Saudi money demand.

This present research claims the empirical contribution in two ways. Firstly, we are estimating a MDF with an asymmetric information of RER utilizing a non-linear Auto-Regressive Distributive Lag (ARDL) technique proposed by Shin et al. [24]. A justification of using a latest non-linear ARDL may be claimed because of a reason that it is utilizing in the very recent monetary and finance literature due to its refined ability to generate the positive and negative changes in the independent variable to test the possible asymmetrical effects on the dependent variable (see [25,26,27]). Further, it can be utilized to find the long run and short run relationships in the model and stability of the model may also be tested afterwards. Secondly, we take advantage of the maximum available annual series of a period 1968–2016. The utilized time period has sufficient number of observations on a real appreciation and depreciation of US dollar (see Fig 1). Therefore, our estimated model may claim for a greater power of generalization than that of all previous studies on MDF of Saudi Arabia.

## Methodology

We start with Pigou’s [28] approach on MDF which is based on the Fisher’s equation of exchange. He explained that money demand has a direct and proportional relationship with nominal income.

$$M_{d}=f(Py)$$ (1)

Where, *M*_*d*_ is for nominal money demand, *P* is a price variable, *y* is for output and (*Py*) represents a nominal income. After incorporating inflation proposed by Bahmani-Oskooee [29] to consider the opportunity cost of holding money and exchange rate proposed by Mundell [4] in money demand function, Eq 1 may be expressed as follows:

$$M_{d}=f(Py,\inf,er)$$ (2)

Where, *M*_*d*_ is money supply, *inf* is showing the inflation and *er* is presenting the exchange rate. Inclusion of US dollar exchange rate in Saudi money demand function is also very relevant as Wen et al. [22] reports that oil price is causing the US dollar exchange rate and Saudi economy is heavily depending on the oil price. Eq 2 is also showing the same model as estimated by a pioneer study of Bahmani-Oskooee [29]. Inflation may be used as opportunity cost of money holding instead of interest rate due to lesser developed financial market of Saudi Arabia. In the presence of less developed financial market, inflation should be used instead of interest rate in the empirical model of MDF [29]. Further, Bahmani [30] also reported the same reason of using inflation for opportunity cost of holding money instead of interest rate for Saudi Arabia. Based on above discussion, our empirical model is as follows:

$$LMD_{t}=f(LGDP_{t},LCPI_{t},LRER_{t})$$ (3)

Where, *t* is a time subscript and is representing a maximum available annual sample time period of 1968–2016. *LMD*_*t*_ is a natural logarithm of nominal money supply (*M3*). *M3* is a proxy for a money demand because of a fact that money demand should be equal to money supply at market equilibrium. *M3* in million Saudi Riyals is sourced from Saudi Arabian Monetary Agency (SAMA). *LCPI*_*t*_ is a natural logarithm of Consumer Price Index (CPI) and it is a proxy for inflation. It is used to consider an opportunity cost of money holding. Money holding may be claimed lesser beneficial with a rising price level because of increasing cost of money holding. Resultantly, people are expected to hold more of real assets instead of liquid money in their portfolio. Therefore, a negative effect of *LCPI*_*t*_ is expected on the money demand. CPI is sourced from WDI. *LGDP*_*t*_ is a natural logarithm of Gross Domestic Product (GDP) and it is a proxy of income level. Increasing income may increase the transaction demand for money due to greater economic activities. Therefore, a positive impact of income is expected on the money demand. GDP in Saudi Riyals is sourced from WDI and has been converted into million Saudi Riyals as *M3* data is also taken in million Saudi Riyals. Chow [31] suggested that nominal income and nominal money demand might be replaced by real income and real money demand respectively if price homogeneity hypothesis could be verified in the MDF. *LRER*_*t*_ is a natural logarithm of Real Exchange Rate (RER). Nominal exchange rate is defined as price of one US dollar in terms of SAR and it is sourced from WDI. Therefore, it is a bilateral nominal exchange rate of US dollar and SAR. It is utilized due a reason that SAR is pegged against US dollar and any movement in US dollar’s value may affect the SAR demand as well [16]. Nominal exchange rate is converted into real by multiplying the CPI of United States and dividing the CPI of Saudi Arabia. CPIs are also sourced from WDI. RER is used instead of nominal exchange rate due to a fact that nominal exchange rate is found as fixed for a long period of time 1987–2016. Further, nominal or real effective exchange rate could not be applied due to unavailability of data for a full range of our sample period and a utility of full range of sample period is very important to have sufficient number of observations on real appreciations and depreciations. A positive movement in RER is showing a real appreciation of dollar and a negative movement is showing a real depreciation of dollar. To avoid confusion, we are not using the terms of the appreciation and the depreciation of SAR. RER may have both positive or negative effects on money demand and the exact relationship is an empirical question. For example, a real appreciation of US dollar may increase the domestic value of foreign assets in SAR and people may demand more SAR by selling foreign assets/currency for a capital gain. Resultantly, a positive relationship may be expected between *LMD*_*t*_ and *LRER*_*t*_. This positive relationship may be termed as a wealth effect. On the other hand, people may expect a further appreciation after a real appreciation of US dollar and may hold/ buy more of the US dollar. Resultantly, demand for SAR may decrease and a negative relationship may be expected between LMD_t_ and LRER_t_. This relation may be termed as a substitution effect / an expectation hypothesis.

Our major objective is to test the possible asymmetrical effects of a real appreciation and a real depreciation of US dollar on the demand for SAR. For this purpose, we are utilizing the Shin et al. [24] methodology and are constructing the following variables:

$$LRER_{t}^{+}=\sum_{i=1}^{t}\Delta LRER_{i}^{+}=\sum_{i=1}^{t}\max(\Delta LRER_{i}^{},0)$$ (4)

$$LRER_{t}^{-}=\sum_{i=1}^{t}\Delta LRER_{i}^{-}=\sum_{i=1}^{t}\min(\Delta LRER_{i}^{},0)$$ (5)

Eqs 4 and 5 divide a variable of *LRER*_*t*_ into *LRER*_*t*_^*+*^ and *LRER*_*t*_^*-*^ by taking the positive and negative partial sums of the changes in *LRER*_*t*_ respectively. Now, *LRER*_*t*_^*+*^ is showing a variable of real appreciation and LRER_t_^-^ is showing a variable of real depreciation of US dollar.

To estimate the Eq 3 after considering asymmetrical effects of RER, we are using the non-linear ARDL methodology of Shin et al. [24]. The choice of this technique is selected due to its efficiency in presence of a mix order of integration as this technique follows the lower and upper bound F-values. In which, lower bound values are provided by assuming level stationary variables i.e. *I(0)* and upper bound values are provided by assuming the first differenced stationary variables i.e. *I(1)*. Most of economic variables are either I(0) or I(1). Therefore, most of studies on MDF using ARDL cointegration test have ignored the unit root analysis [30,10,5,6,8]. Most importantly, this methodology allows us to test the asymmetrical effects of *LRER*_*t*_^*+*^ and *LRER*_*t*_^*-*^ on the demand for SAR. The non-linear ARDL model of our hypothesized MDF is as follows:

$$\begin{array}{l} \Delta LMD_{t}=\gamma_{0}+\gamma_{1}LMD_{t-1}+\gamma_{2}LCPI_{t-1}+\gamma_{3}LGDP_{t-1}^{}+\gamma_{4}LRER_{t-1}^{+}+\gamma_{5}LRER_{t-1}^{-}\\ +\sum_{j=1}^{q1}\delta_{1j}\Delta LMD_{t-j}+\sum_{j=0}^{q2}\delta_{2j}\Delta LCPI_{t-j}+\sum_{j=0}^{q3}\delta_{3j}\Delta LGDP_{t-i}^{}\\ +\sum_{j=0}^{q4}\delta_{4j}\Delta LRER_{t-1}^{+}+\sum_{j=0}^{q5}\delta_{5j}\Delta LRER_{t-1}^{-}+\xi_{t} \end{array}$$ (6)

Where, *q1*, *q2*, *q3*, *q4* and *q5* are optimum lag lengths selected by Akaike Information Criterion (AIC) after imposition 2 maximum lags for each variable. We avoid more than 2 lags to save the degree of freedom. Moreover, Perron [32] suggested to include maximum 4 lags for high frequency data like quarterly data and maximum 2 lags for low frequency data like annual data in our case. Following this idea, latest study on Saudi MDF by [13] has also been utilized maximum 2 lags using annual data. After deciding optimum lag length, Eq 6 may be tested for a cointegration on a null hypothesis, *γ*_1_ = *γ*_2_ = *γ*_3_ = *γ*_4_ = *γ*_5_ = 0 and long run effects of LCPI_t_, LGDP_t_, LRER_t_^+^ and LRER_t_^-^ may be assessed by normalizing the *γ*_2_, *γ*_3_, *γ*_4_ and *γ*_5_ respectively by *γ*_1_. After estimating the coefficients of LRER_t_^+^ and LRER_t_^-^, the evidence of asymmetry can be tested through Wald test on null hypothesis of symmetry (H_0_: −γ4γ1=−γ5γ1). A rejection of H_0_ may be claimed for the presence of asymmetry. Further, the price and income homogeneity hypotheses may be tested by imposing a restriction (elasticity = 1) on the long run coefficients of price and income respectively. Further, the lagged level variables can be replaced with the lag of Error Correction Term (ECT_t-1_) for an evidence of short run relationship in the model and short run effects can be captured through estimated parameters of differenced variables in the Eq 6.

## Results and discussions

**Fig 2:**
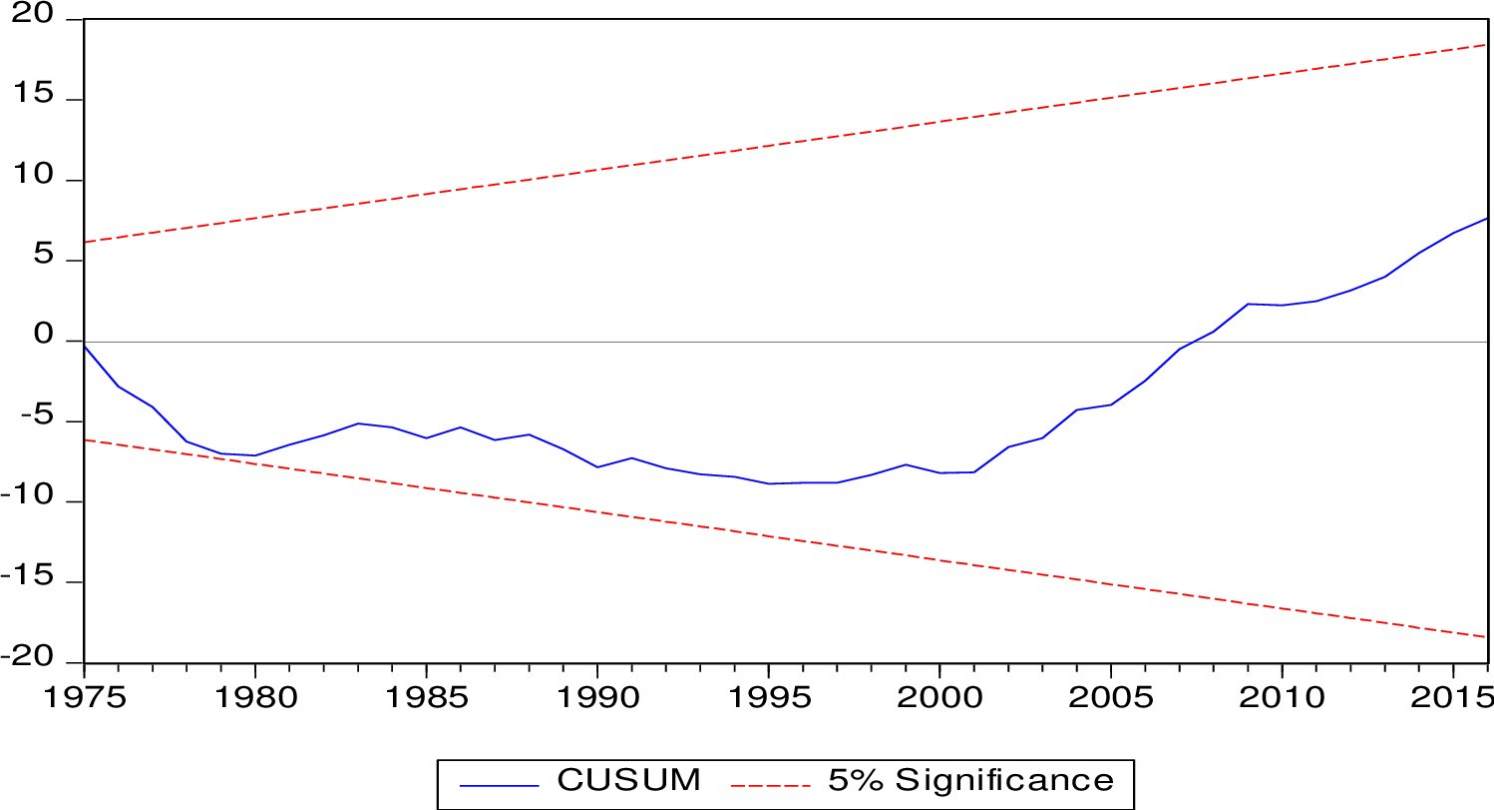
CUSUM test.

**Fig 3:**
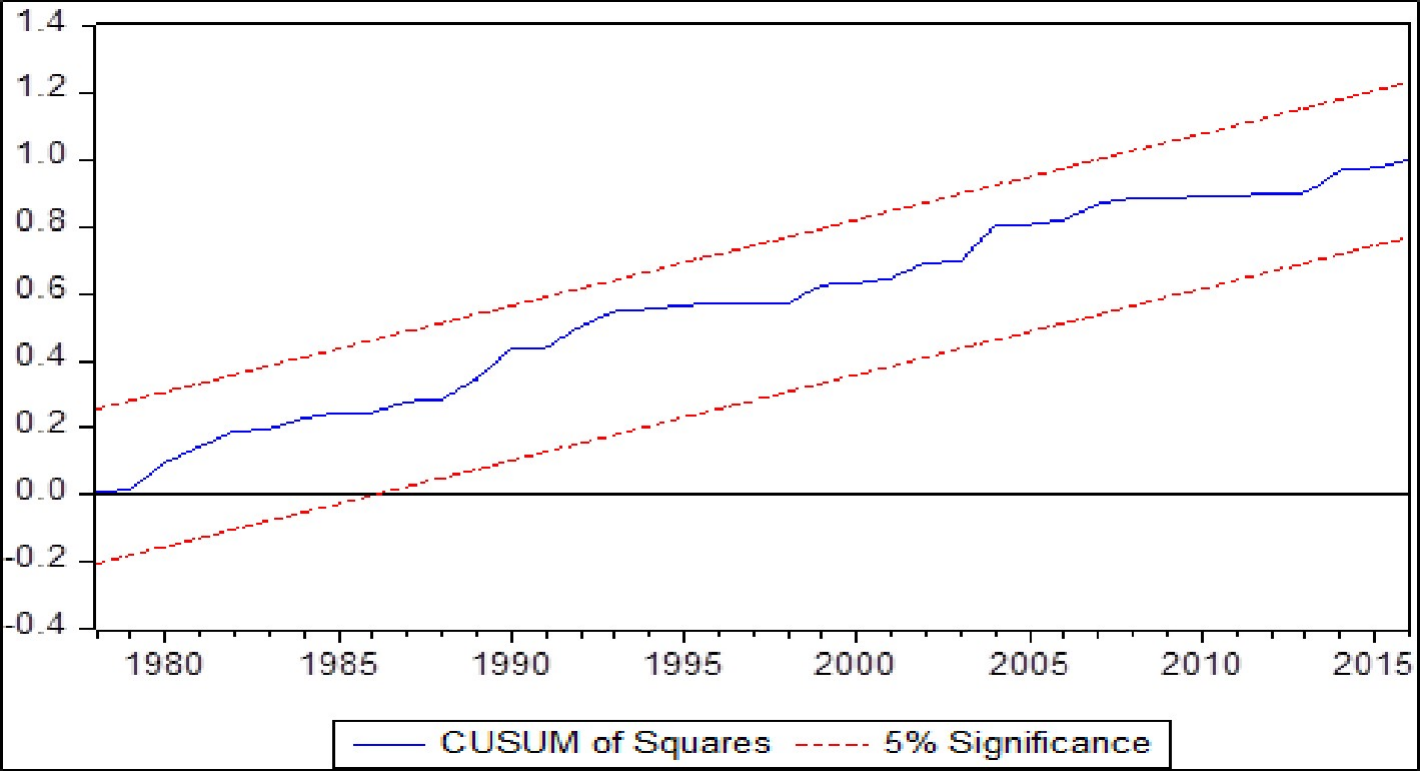
CUSUMsq test.

Table 1 is showing the results from non-linear ARDL model mentioned in the Eq 6 after selection of optimum lag length through AIC and after inclusion of two dummy variables. The dummy variables are incorporated to estimate the effect of a very sharp decline in real exchange rate in the year 1971 (D1) and to capture the effect of a recent financial crisis of 2008 (D2). Dummy variables assume zero before the years 1971 and 2008 for D1 and D2 respectively and one afterwards. The diagnostic tests are showing the low F-values and reasonability high probability values. Therefore, the estimated Eq 6 may be claimed free out of heteroscedasticity, serial correlation and non-normality of error term and also free from the misspecification of functional form. The estimated F-value, from bound test on the null hypothesis of no-cointegration, is 17.4786. This estimated F-value is higher than upper critical bound F-value of Narayan [33] at 1% level of significance. Therefore, we can claim a cointegration in the Eq 6. Further, the estimated CUSUM and CUSUMsq tests’ values presented in Figs 2 and 3 are within critical values. Therefore, a stable MDF can be claimed and money supply as an instrument of monetary policy may be recommended to the economy of Saudi Arabia as suggested by Poole [1].

**Table 1:** Money demand function.

Variable	Parameters	S.E.	t-Statistic	p-value
Long Run Results				
LCPI_t_	-3.5051	1.2716	-2.7564	0.0091
LGDP_t_	1.3036	0.2307	5.6515	0.0000
LRER_t_^+^	0.4638	0.2539	1.8270	0.0760
LRER_t_^-^	-4.6387	1.3928	-3.3304	0.0020
D1	-0.5820	0.3513	-1.6566	0.1063
D2	-0.0593	0.1375	-0.4310	0.6691
Intercept	6.4739	2.4138	2.6820	0.0110
Wald Test (H_0_:Symmetry)	F-value = 15.2719		p-value = 0.0001	
Short Run Results				
ΔLCPI_t_	-0.9986	0.2724	-3.6658	0.0008
ΔLGDP_t_	0.2256	0.0319	7.0777	0.0000
ΔLRER_t_^+^	-1.0079	0.3489	-2.8884	0.0065
ΔLRER_t_^-^	-1.7011	0.3520	-4.8329	0.0000
D1	0.0001	0.0493	0.0016	0.9988
D2	-0.0169	0.0364	-0.4636	0.6457
ECT_t-1_	-0.2849	0.0647	-4.4026	0.0001
Wald Test (H_0_:Symmetry)	F-value = 2.0051		p-value = 0.1568	
Diagnostics				
Bound Test	Estimated F-value = 17.4786			
Level of Significance	Lower Critical Value		Upper Critical Value	
At 10%	2.372		3.320	
At 5%	2.823		3.872	
At 1%	3.845		5.150	
Heteroscedasticity	F-value = 0.9484		p-value = 0.5079	
Serial Correlation	F-value = 0.9395		p-value = 0.4008	
Normality	F-value = 1.5397		p-value = 0.4631	
Functional Form	F-value = 0.9318		p-value = 0.3410	

In the long run results, *LCPI*_*t*_ is showing a negative impact on the demand for SAR and this result proofs that a rising inflation forces people to demand lesser riyals and more of other real assets. It means that rising inflation is rising the opportunity cost of holding SAR. Consequently, people are preferring real assets in their portfolio instead of SAR. This result is corroborating the past studies’ results of AlKaswani and Al-Towaijari [17], Bahmani [30] and Mahmood and Asif [20]. Further, price homogeneity hypothesis (elasticity = 1) has been tested and rejected with estimated Chi-square value of 22.20 and p-value of 0.00. Therefore, the selection of nominal money supply and nominal income in our MDF is more appropriate as suggested by [31]. The coefficient of *LGDP*_*t*_ is found positive, greater than one and significant. Therefore, a rising income is increasing the transaction demand for SAR. This result is in line of all past studies’ results except the insignificant effect of income in a study of Lee et al. [19]. Further, income homogeneity hypothesis (elasticity = 1) has been tested and rejected with estimated Chi-square value of 4.3033 and p-value of 0.038. Therefore, we may conclude money as a luxury good in case of Saudi Arabia as estimated elasticity is found larger than one.

Basically, non-linear ARDL has been applied to test the possibility of asymmetry in the effects of a real appreciation and a real depreciation of US dollar on the demand for SAR. In this context, Wald test on the null hypothesis of symmetry is rejected at 1% level of significance. Therefore, we may claim the existence of asymmetrical effects of LRER_t_^+^ and LRER_t_^-^ in the long run. Further, the coefficients of LRER_t_^+^ and LRER_t_^-^ have different signs. This result is corroborating the different direction of effects of the depreciation and the appreciation on the Iran’s money demand reported by Bahmani-Oskooee and Bahmani [5]. In our results, a real appreciation of US dollar (the coefficient of LRER_t_^+^) is showing a positive and significant effect on the demand for SAR. This result is following the results of previous studies on testing the symmetrical effect of exchange rate [17,30,18,19,34]. These mentioned studies have used the sample time periods of Saudi Arabia in which a real appreciation of US dollar was more dominant. Therefore, the previous studies which ignored the real depreciation of US dollar during the pre-1978 period or the post-2006 period or both are in line with our asymmetrical effect of a real appreciation of US dollar. Because, these studies have utilized a less number of observations of a real depreciation in the symmetrical analysis. A positive effect of a real appreciation of US dollar is corroborating the existence of wealth effect. It means that the value of foreign assets in terms of SAR increases with a real appreciation of US dollar. Resultantly, people are selling the foreign assets/currency for a capital gain and demand for SAR is increasing. This effect of a real appreciation is also corroborating the activities of speculation in the developed cities of Saudi Arabia.

On the other hand, the coefficient of LRER_t_^-^ is showing a negative impact on the demand for SAR. This result is following the result of Al Rasasi [16] who used a balance number of observations of appreciation and depreciation in the estimated model. Further, our result is opposing the symmetrical positive effect of exchange rate reported by all of past literature on Saudi Arabia except Al Rasasi [16]. As mentioned above, previous studies ignored the pre-1978 period or post-2006 period or both which have consecutive real depreciation of US dollar. However, our sample period is covering maximum available observations of both of pre-1978 and post-2006 periods. Therefore, our estimated negative effect of a real depreciation of US dollar is more reliable and has greater generalization power than the previous studies. Further, our estimated negative effect of a real depreciation is showing the existence of a substitution effect/an expectation hypothesis. It means that a real depreciation of US dollar is raising the expectations of further depreciation. Resultantly, demand for dollar is decreasing and demand for SAR is increasing. The existence of substitution effect is also corroborated from the data series of RER as a real depreciation of US dollar has been observed persistently for the two long time spans of 1971–1977 and 2007–2016 in our sample period. Therefore, investors may predict the further depreciation once US dollar is depreciated. The presence of substitution or expectation hypothesis in the estimations may also be matched with oil price movements. A consistent rise in oil price has been observed during the above mentioned periods 1971–1977 and 2007–2016 except declines in the years 2009 and 2013–16. Rising oil price is good news for an oil-based economy of Saudi Arabia and may generate higher export revenues and economic growth. It is because of a reason that oil exports are constituted more than 80% of total exports. Therefore, a consistent real appreciation (depreciation) of SAR (US dollar) may also be expected because of higher oil exports’ earnings. In response, speculators are selling the US dollars from their portfolio and are demanding the SAR. Moreover, the coefficients of dummy variables are statistically insignificant. Therefore, the selected structural breaks have statistically insignificant effects on the demand for SAR.

The short run relationship in our model is corroborated with a negative and significant coefficient of *ECT*_*t-1*_. The coefficient of Δ*LCPI*_*t*_ is showing a negative and significant effect of inflation on the demand for SAR in the short run. The rising inflation is also increasing opportunity cost of holding money in the short run. Therefore, the demand for SAR is decreasing and real assets are demanded instead. The coefficient of Δ*LGDP*_*t*_ is positive and significant and is showing the higher transaction demand for SAR with a higher level of income in the short run. The directions of positive (negative) effects of income(inflation) are same in the short and long runs but magnitude of effects are significantly decreased in the short run. Further, Δ*LRER*_*t*_^*+*^ variable is showing a negative and significant effect on the demand for SAR and is corroborating an existence of substitution effect of a real appreciation in the short run. This means that a real appreciation of US dollar is raising the expectations of further appreciation. Resultantly, the demand for dollar is increasing and the demand for SAR is decreasing. The substitution effect is also hold due to a fact that people may consider dollar dearer with a real appreciation of dollar and may consider SAR inferior in the short run. The coefficient of Δ*LRER*_*t*_^*-*^ is showing a negative effect on the money demand like a negative effect of a real appreciation in short run and is also corroborating a substitution effect. The negative effect of Δ*LRER*_*t*_^*-*^ is showing that a real depreciation of dollar may develop the expectation of further depreciation. Therefore, people are considering SAR dearer with a real depreciation of dollar and US dollar inferior in their portfolio. Resultantly, they are demanding more of SAR. Both Δ*LRER*_*t*_^*+*^ and Δ*LRER*_*t*_^*-*^ are showing negative effects (expectation hypothesis) on the demand for SAR in short run and Wald test is also proofing the symmetrical effects of Δ*LRER*_*t*_^*+*^ and Δ*LRER*_*t*_^*-*^ on the money demand. A negative symmetrical effect of exchange rate in the short run may be claimed due to a reason that a consistent appreciation or a consistent depreciation may be observed more prominent in the short run period. Therefore, any positive or negative movement in the exchange rate may develop the expectation of the same movement in the future. Resultantly, expectation hypothesis is dominant over wealth effect for both positive and negative movement of exchange rate in the short run.

## Conclusions and recommendations

The accurate estimation of MDF and testing its stability are very important for monetary policy perspectives. Particularly, the inclusion of exchange rate in the MDF is more interesting in integrating the monetary and exchange rate policies. The effects of income, inflation and RER have been tested on the demand for SAR in Saudi Arabia using a non-linear ARDL model and using a long time period 1968–2016. The presence of long and short relationships in the MDF has been corroborated in the empirical testing. Inflation has been negatively impacted the demand for SAR in the both long and short runs. This negative effect is confirming that inflation is showing a rising cost of holding liquid money and is also showing a preference of holding real assets in the time of inflation. Further, price homogeneity hypothesis has been rejected for the estimated elasticity in the long run. Income has been positively affected the transaction demand for SAR in the both long and short runs. Moreover, income homogeneity hypothesis has been rejected for the estimated elasticity greater one in the long run. Therefore, we may conclude money as a luxury good in the long run. The different signs and Wald test have confirmed the existence of asymmetrical effects of the real appreciation and of the real depreciation of US dollar on the demand for SAR in the long run. A real depreciation of US dollar has confirmed a substitution effect. Therefore, a real depreciation of dollar may develop the expectations of further depreciation in the long run. Resultantly, the demand for dollar is decreasing and the demand for SAR is increasing. Conversely, a real appreciation of US dollar has corroborated the existence of wealth effect in the long run. It is increasing the domestic value of foreign assets/currency. Resultantly, people are selling foreign assets/currency for a capital gain and are increasing the demand for SAR in the long run with a real appreciation of US dollar. This is also confirming the habits of people in developed cities to speculate the currencies for a capital gain. Based on the results, it is concluded that demand for SAR is increasing with both real appreciation and real depreciation of US dollar in the long run. In large, we may reject the symmetrical effect of exchange rate on the demand for SAR reported by past literature on the Saudi Arabia in the long run. However, a real appreciation and a real depreciation of US dollar are showing negative symmetrical effects on the demand for SAR and are corroborating an existence of a substitution effect in the short run.

The MDF is found stable through CUSUM and CUSUMsq tests. Therefore, we may recommend the monetary authorities of Saudi Arabia to use the money supply as a monetary policy instrument. Further, a higher money demand is a symbol of a higher economic activities in the country. Therefore, Saudi government should control inflation as it has a negative effect on the demand for SAR. Recently, inflation has been observed due to indirect taxes imposed by Saudi government and it may have adverse effect on the demand of SAR and on the overall economic health of the country. Moreover, our results corroborate that demand for SAR is increasing with both appreciation and depreciation in the long run. Therefore, the supply of SAR should be increased to meet the higher demand with any change in the RER. Lastly, estimated elasticities of non-linear ARDL may be utilized by monetary authorities to supply the right amount of SAR for any movement of income, inflation and real exchange rate.

## Supporting information

S1 File Data series. (XLSX)
